# Supplementary material for: Patterns of Intron Gain and Loss in Fungi
Source: PLoS Biol. 2004 Nov 30;2(12):e422. doi: 10.1371/journal.pbio.0020422 (PMC532390; doi:10.1371/journal.pbio.0020422)
Supplement: Table S1 — Also available at http://genes.mit.edu/NielsenEtAl/. (4.3 MB ZIP). [file pbio.0020422.st001.zip › NielsenEtAl/html/964.html]

AN3716.1.NCU02374.1.MG03524.1.FG07956.1


```
 CLUSTAL W (1.82) Multiple Sequence Alignments - Introns Inserted


Sequence 1: NCU02374.1	381 aa
Sequence 2: MG03524.1	382 aa
Sequence 3: FG07956.1	381 aa
Sequence 4: AN3716.1	381 aa
Alignment Length: 382 aa
Number Identitical Residues: 201 aa
Alignment Score (without introns) 10128


MG03524.1 	MSADTVADFLADQRDDAPEELQPLILKFEDYWERKLWHQLTEASLEFFDHPISRPARLHF
NCU02374.1	MNVDTISDFLAEQRDQAPEELQPLVLEFETLWERKLWHQLTNLLLEFFNHPGSAPQRLQF
FG07956.1 	MSNDAIADFLSEQRDEAPEELQPLILDFENFWERKLWHQLTDALIDFFSHPESAPQRLQF
AN3716.1  	MENQKISDFLADQLQQAPEQCQASFLSFEEYWERKLWHQLTNALIDFFRLPESAPQRLPL
          	*. : ::***::* ::***: *. .*.**  **********:  ::**  * * * ** :

MG03524.1 	YKVFVAKFADKINQLKLVDIALKAATAAKD1PKERLQFLETVAKKVDDEDSQDAYVFALI
NCU02374.1	YKVFILKFADKINQLKLVDLALKAATQCRD1NQERLSFLEAVVKKVDHEDSQDAYVFATI
FG07956.1 	YRVFILKFADKINQLKLVDLALKAATECDE1EEERLSFLQSVAKKVDNENSQDAYVFALV
AN3716.1  	FKTFVLSFADKINQLNFVSLGLMASTECAD1DQERLSFLTSLADKVNKPDSQDAYIYALA
          	::.*: .********::*.:.* *:* . :  :***.** ::..**:. :*****::*  

MG03524.1 	AVARVKLDLKDPEGTRKDLDKAERILDSFDSVETVVSAAFYSAQADYYQ~AQADFASYYR
NCU02374.1	AVARVKLDLSDLDGARKDLDTAERILDNFDSVESVVHAAFYDTNAKYFQ~AKSDWGSYYK
FG07956.1 	AVAQAKLDLEELDGARKDLDTAERILDSFDSVENVVHAAFYDANAIYYQ0RKMDFSNYYR
AN3716.1  	EVANVKQRLHNLDGAQKDLETCQKVLDSFDAVENVVHASFYKVNADYYH0AKQEFASFYK
          	 **..*  * : :*::***:..:::**.**:**.** *:**..:* *::  : ::..:*:

MG03524.1 	NALLYLACIDVSALTPEERRSRAYDLAIAALASTSIYNFGELLLHPILDALTLSDKDSWL
NCU02374.1	NALLYLACIDVKSLSLEEQRQRAYNLAIAALVSSSIYNFGELLLHPILDALTIDDRHKWL
FG07956.1 	TALLYLACIDINELSPDERHKRAYHLSIAALVSTSIYNFGELLLHPILDVLSKSE-HSWM
AN3716.1  	NALLYLACIDLEDLSESERVSRAYNLSVAALVSDSIYNFGELLLHPILDSLTETP-HSWL
          	.*********:. *: .*: .***.*::***.* *************** *:    ..*:

MG03524.1 	RELLFAFNRGDLAAYDVLSGHIGSNPLLAKHRGQLRQKIYLAALTEAVFRRPPHDRAMTF
NCU02374.1	HQLLFAFNRGDLAAYDLLASHISSDPLLAQHTRQLREKIYLAALTEAVFRRPPHDRAMSF
FG07956.1 	RDLLFAFNRGDLAAYDQLSDRIDSHKLLKSNSTHLRQKIYLSALTEAVFRRAPHDRTLTF
AN3716.1  	RDLLFAFNRGDLTAYDVLAGNISKNQLLQAHRFFLYQKISLSALTEMVFRRPPHDRNLTF
          	::**********:*** *:..*... **  :   * :** *:**** ****.**** ::*

MG03524.1 	QTIARETKVQPDEIEHLVMKALSLGLLRGNIDQVDEVAHINWVQPKVLDMKQIDNMRLRL
NCU02374.1	STIANETKVHPDEIEHLIMKALSLDLLRGTIDQVDEVAHINWVQPKVLDMQQIKNMRQRL
FG07956.1 	ATIAQETKVRPEEIEHLIMKALSLGLLRGTIDQVDGVAQITWVQPKVLDMKQIAAMRQRL
AN3716.1  	ASISAETKVKPEEIEHLVMKALSLGLLKGAIDQVGQIAQINWVQPKVLDMKQIEGMRNRL
          	 :*: ****:*:*****:******.**:* ****. :*:*.*********:**  ** **

MG03524.1 	KDWDSSVNQLGNWIEGVGQDVWAV
NCU02374.1	QEWDSSVDKLGNWIEGVGQDVWA-
FG07956.1 	LDWDSSVNQLGNWIESAGKDVWAA
AN3716.1  	KDWDAGVNQLGHWIEGVGKDVWAA
          	 :**:.*::**:***..*:****
```
